# Innovation in Acute Adolescent Mental Healthcare: Evaluating a Responsive and Sustainable Mental Healthcare Model

**DOI:** 10.3390/healthcare14172837

**Published:** 2026-09-03

**Authors:** Anjana Thazhissery, Md Nazmul Huda, Antonio Mendoza Diaz, Ingrid Bartrop, Natalie Cabrera, Valsamma Eapen, Rajeev Jairam

**Affiliations:** 1 Gna Ka Lun Inpatient Unit, Campbelltown Hospital, South Western Sydney Local Health District, NSW Health, Campbelltown, NSW 2560, Australia; anjana.thazhissery1@health.nsw.gov.au (A.T.); info@psychologytrainingandsupervision.com.au (N.C.); v.eapen@unsw.edu.au (V.E.);; 2 Infant, Child and Adolescent Mental Health Service, South Western Sydney Local Health District, NSW Health, Sydney, NSW 2170, Australia; ingrid.bartrop@health.nsw.gov.au; 3 Discipline of Psychiatry and Mental Health, School of Clinical Medicine, University of New South Wales, Sydney, NSW 2052, Australia; 4 Academic Unit of Infant, Child, and Adolescent Psychiatry Services, Ingham Institute for Applied Medical Research, Liverpool, NSW 2170, Australia; 5 Tasmanian Centre for Mental Health Service Innovation, Tasmanian Health Service, Hobart, TAS 7000, Australia; tony.mendoza.diaz@ths.tas.gov.au; 6 School of Medicine, Western Sydney University, Campbelltown, NSW 2560, Australia

**Keywords:** innovative model of care, effectiveness, child and adolescent mental health, inpatient unit, outcomes

## Abstract

**Background and objectives:** Despite the substantial global burden of mental illnesses, there remains limited evidence evaluating the effectiveness of inpatient Models of Care (MOCs) for adolescents. This paper compares the effectiveness of an innovative MOC, the Brief Intervention MOC (BIMOC), with the Traditional MOC in an acute adolescent inpatient unit. Comparisons were based on clinical outcome measures and several Key Performance Indicators (KPIs) (length of stay, 28-day readmission rate, 7-day follow-up rate, seclusion rate, and self-harm incidents), which were tracked over the subsequent years to evaluate BIMOC’s effectiveness and sustainability. **Materials and Methods:** A retrospective, single-center, quasi-experimental non-randomized before-and-after study using historical controls was conducted. Clinical outcomes were assessed using the Health of the Nation Outcome Scales for Children and Adolescents (HoNOSCA) and the Children’s Global Assessment Scale (CGAS). Service-level KPIs were compared across models. Descriptive statistics, paired-samples *t*-tests, Cohen’s d, and z-tests were used to evaluate changes within models and compare outcomes between models. **Results:** Within the BIMOC, both acute and crisis subgroups showed substantial improvements, with mean CGAS scores increasing from 27.67 to 49.76 and mean HoNOSCA scores decreasing from 19.30 to 9.96 (*p* < 0.001). Overall, both models demonstrated significant improvements from admission to discharge. Effect size analysis indicated greater functional gains under the BIMOC compared with the TMOC (d = 1.89 vs. 1.22; z = −3.694, *p* = 0.0002), while no statistically significant difference in symptom improvement was detected between the two MOCs (z = −0.769, *p* = 0.4418). KPI data analysis identified favorable patterns in several service-level indicators during the BIMOC period compared to the TMOC period, including reduced length of stay (21.0 vs. 13.6 days), reduced 28-day readmission rates (11.9–17.0% vs. 8.1%), and increased 7-day follow-up completion (approximately 60% vs. 76.8%). **Conclusions:** The study suggests that a structured and integrated inpatient MOC (BIMOC) incorporating multidisciplinary collaboration, family involvement, and coordinated discharge and follow-up processes may support positive clinical and service-level outcomes in adolescent mental health inpatient settings. However, given the observational before-and-after design, the findings should be interpreted cautiously and do not establish causality.

## 1. Introduction

Mental health conditions frequently emerge during childhood and adolescence, and they are related to a higher risk of adult mental disorders [1]. This is a global health concern, with profound effects on families and communities, as about one in seven adolescents will experience serious mental health problems [2], accounting for an estimated 14% of the total burden of disease [3]. While the majority of adolescents with mental health problems are cared for in the community, inpatient care is indicated particularly when there is a need for intensive interventions that cannot be provided in the community, such as when there is an increased risk of harm to self or others or when the young person presents with a deterioration in physical health as a result of mental health difficulties [4].

The models of care in acute adolescent inpatient units around the world are diverse [5], and the models are shaped by a myriad of factors, such as mental health literacy, care pathways, stigma, extent of family engagement and integration, and availability of follow-up arrangements [6], staffing profiles, resource availability [7], and differing thresholds for admission. In Europe, the acute adolescent inpatient unit is part of a continuum-of-care model linked to outpatient treatment and community-based services [8]. The Child and Adolescent Mental Health Service (CAMHS) is delivered through a tiered model of service organization with tier 4 being the highest tier of severity and complexity, involving highly specialized inpatient care funded by the government [9]. In 2017, a United Kingdom-based study examining the outcomes of inpatient psychiatric admissions for adolescents provided evidence to support the efficacy of a tier-4 service [10]. In the United States, the model of care has gradually evolved from institutional and fragmented service delivery toward more community-based and integrated models, although fragmentation has remained a persistent problem [11]. Although over half a million adolescents receive mental health services through inpatient psychiatric care [12,13], the evidence base evaluating the effectiveness of inpatient care for adolescents with mental health problems is limited.

In Australia, the vast geographic landmass creates differences in population distribution across urban, regional, remote, and very remote areas, requiring innovative service development. With the paucity of emergency, residential, day-patient and in-home services for adolescents and their families, there is growing pressure on inpatient units to admit patients for clinical reasons as well as for non-clinical reasons of respite and primary child protection issues [4]. Thus, inpatient care consumes the majority of child and adolescent mental health resources, and as societal mental health demands increase, many inpatient services have been encouraged to reduce costs and to justify the spending via evidence on outcomes and effectiveness [12,14] as well as expectations to increase efficiency and cater to more patients within their existing pool of funds [12]. Therefore, there is a need to examine valid and reliable outcome measures that capture changes in symptoms and functioning, and which determine the effectiveness of inpatient psychiatric admissions for adolescents. These measures provide a means for the evaluation of an inpatient service as a whole and highlight areas that warrant improvement [15]. Subsequently, all states and territories have implemented routine outcome measurements (ROMs) across public mental health services. Core measures in the adolescent setting include the Health of Nations Outcome Scales for Children and Adolescents (HoNOSCA) [16] and the Children’s Global Assessment Scale (CGAS) [17].

In many high-income countries, healthcare systems use performance indicators or Key Performance Indicators (KPIs) to monitor, measure, and manage the performance of their healthcare systems to ensure effectiveness, efficiency, and equity [18]. The Donabedian conceptual model provides a framework for evaluating healthcare services and quality of care. KPIs are typically organized according to the Donabedian Framework, which includes the structure, process, and outcome domains [19]. Structure describes the context in which healthcare is delivered; process includes all transactions between patients and providers throughout the delivery of care; and outcome refers to the effect of healthcare on the health status of patients and the population [19]. In Australia, the National Mental Health Performance Framework (NMHPF) is a key strategy for facilitating a culture of continuous quality improvement in mental health service delivery [20]. The framework supports Australian state and territory governments’ commitment to improving accountability and transparency at the Mental Health Service Organization (MHSO) level. The NMHPF follows a tiered, domain-based structure, with tier 3 dedicated to health system performance, as monitored via KPIs. The KPIs reflect an inpatient unit’s efficacy, effectiveness and safety through metrics of the length of stay (LOS), 28-day readmission rate, rate of seclusion and restraint, and 7-day post-discharge follow-up rate [21]. However, few studies have examined these KPIs to assess the effectiveness of MOCs.

Taken together, there is little published literature about effective models of care in adolescent mental health inpatient units. Moreover, to our knowledge, there are few studies in Australia that compare different models of care using outcome measures and KPIs. The aim of this study was to evaluate the effectiveness of an innovative model of care, the Brief Intervention Model of Care (BIMOC), compared with the Traditional MOC (TMOC) in the acute adolescent inpatient unit. The findings are expected to contribute to improving the quality of mental healthcare for young people.

## 2. Materials and Methods

### 2.1. Design

This study employed a quasi-experimental, non-randomized before-and-after study design using an Electronic Medical Records (EMR)-based file audit of routinely collected data from consumers, their parents, and carers attending the BIMOC and TMOC services. The study was supplemented by longitudinal service-level KPI data to evaluate the sustainability of outcomes over time. Outcomes under the TMOC (2016–2017) were compared with outcomes following implementation of the BIMOC (2018). The sustainability of BIMOC was further examined using KPI and clinical data available from 2022 to 2025. Planning and preparation for BIMOC commenced during late 2017, including consultations with clinicians and the development of implementation procedures. However, BIMOC was not introduced into clinical practice until January 2018. Consequently, all admissions occurring during 2016–2017 were managed under the TMOC and were included within the TMOC cohort, whereas all admissions from January 2018 onwards were managed under the BIMOC service framework and were included within the BIMOC service-framework cohort. The BIMOC was implemented as a routine clinical model of care within the inpatient service and was not introduced as a research intervention. The present study was a retrospective evaluation of routinely collected clinical data generated during standard care delivery. TMOC served as the historical comparator, while BIMOC became the routine model of care thereafter. The study is therefore considered quasi-experimental because outcomes were compared before and after implementation of a service-level intervention without random allocation.

### 2.2. Site Descriptions

The inpatient unit Gna Ka Lun (GKL) (indigenous word meaning “healing of mind”) was based within the Campbelltown hospital premises of South Western Sydney. It covers a wide, densely populated geographical region, including both metropolitan and rural communities. GKL is a 10-bed acute adolescent unit established in the early 2000s, with an evidence-based care model that aligned with similar inpatient units. Prior to 2017, the inpatient unit provided a traditional model of care (TMOC) based on standard psychiatric assessment, treatment, and discharge planning processes. A detailed description of a TMOC is provided in Section 2.3.

Apart from the implementation of the Brief Intervention Model of Care (BIMOC) in 2017, there were no major changes in ward infrastructure, bed capacity, staffing levels, multidisciplinary team composition, admission criteria, or referral pathways during the study period (2016–2025). The BIMOC remained the standard model of care and was implemented consistently throughout this period.

### 2.3. Brief Intervention Model of Care (BIMOC) and TMOC

A traditional MOC represents a conventional, non-stratified acute inpatient psychiatric care model delivering standard assessment, treatment, and discharge planning without structured, time-limited or differentiated intervention pathways. The BIMOC is a structured, time-limited psychosocial intervention designed for adolescents presenting in mental health crisis, typically delivered over 2–5 sessions during hospital admission. The model aims to stabilize the immediate crisis, enhance coping skills, develop an individualized care plan, strengthen family involvement, and facilitate continuity of care following discharge. The BIMOC was developed after analyzing the pre-existing TMOC, relevant legislative requirement for service delivery and contemporary evidence-based practice. The model was informed by the Clinical Practice Guideline for the Management of Borderline Personality Disorder (NHMRC) and the Project Air Strategy Brief Intervention Manual and Adolescent Intervention Guide for Clinicians [22,23]. These resources emphasized the importance of brief, targeted interventions, minimizing unnecessary hospital admissions and lengths of stay, and promoting continuity of care through coordinated psychosocial support. Within 24–48 h of admission to the inpatient unit, a comprehensive diagnostic and psycho-social assessment of the adolescent and their family/carers was conducted by a multidisciplinary team (MDT) led by a Consultant Psychiatrist, a Psychiatry Registrar, a Clinical Psychologist, a Social Worker, and a Nurse. The classification process was based on a structured clinical assessment incorporating psychiatric diagnosis, presenting risks, psychosocial context, treatment history, and current service involvement. No formal diagnostic instrument was used specifically for classification; rather, allocation was determined through MDT consensus informed by clinical assessment and available clinical records. Following the assessment, the MDT developed a biopsychosocial formulation and classified each adolescent as either a “crisis” or an “acute” admission according to predefined clinical criteria and treatment pathways (Supplementary Materials Section S1). Acute admissions typically included adolescents with enduring mental disorders, such as Major Depressive Disorder unresponsive to community treatment, Bipolar Affective Disorder, early-onset psychosis (including schizophrenia), severe treatment-resistant Obsessive Compulsive Disorder, and Post-Traumatic Stress Disorder (PTSD), who presented with deterioration in mental health and risk levels that could not be safely managed through community-based interventions alone. Crisis admissions usually comprised adolescents presenting with acute emotional dysregulation, escalating self-harm risk, suicidal ideation or suicide attempts, absconding behavior, or sexual safety concerns, usually arising in the context of psychosocial stressors, situational crises, and/or trauma reactivation. A significant proportion of adolescents in this group had limited or no prior engagement with community mental health services. The resulting care plan identifies the primary treating team, outlines the goals of treatment, the length of stay, the estimated date of discharge, and the schedule for family meetings and individual sessions with the admitted patient. The care plan is shared with the adolescent, family/carers, and community team to support collaborative care and continuity following discharge.

A bespoke model was developed for young people admitted with ongoing suicidal ideation, self-harm, emotional dysregulation, and interpersonal difficulties. The model emphasized rapid engagement following a mental health crisis, collaborative development of an individualized care plan, identification of triggers, warning signs, protective factors, and coping strategies, psychoeducation regarding emotion regulation and crisis management, goal-setting informed by the adolescent’s strengths and values, active involvement of families and carers, and facilitation of a transition to community-based mental health services. A brief intervention is offered to those identified as crisis admissions. This is a time-limited, goal-oriented, patient- and family-centered, culturally sensitive intervention. The components in this model (Supplementary Materials Section S2) include nominating a ‘therapeutic lead’, agreeing upon targeted family assessments, brief interventions based on trauma-informed care, collaborating with the adolescent to develop self-harm prevention strategies, community CAMHS integration, and timely, well-communicated discharge planning [24]. In addition, skills training is provided for the adolescent and the family, while discharge planning is developed in collaboration with the adolescent, the family and the community team. The BIMOC integrates principles from dialectical behavior therapy (DBT), cognitive-behavioral approaches, family-focused interventions, and trauma-informed care, with the primary aim of reducing future crises, improving emotion regulation, strengthening family support, and promoting continuity of care following discharge. This approach aims to break down barriers to adolescents engaging with the service, whilst reducing the rate of seclusion and minimizing the ‘revolving-door’ phenomenon of repeated hospital presentations. Although the brief intervention component was delivered specifically to adolescents classified as crisis admissions, all adolescents admitted following the implementation of the BIMOC were managed within the broader BIMOC service framework, including structured multidisciplinary assessment, treatment planning, family engagement, and coordinated discharge planning. Therefore, all admissions occurring during the BIMOC period were included within the BIMOC service-framework cohort for service-level evaluation purposes. The adolescents categorized as ‘acute’ received standard treatment components consistent with the TMOC; however, they remained within the broader BIMOC service framework, including structured multidisciplinary assessment, care planning, family involvement, and discharge coordination.

### 2.4. Study Population and Sampling

All adolescents aged 10–18 years admitted to the GKL unit between January 2016 and December 2018 and between January 2022 and December 2025 were included in this study (2016 and 2017 (TMOC) and 2018 (BIMOC); both were run throughout from 2022 to 2025). Admissions during 2016–2017 were classified as receiving the Traditional Model of Care (TMOC). Although planning for Brief Intervention Model of Care (BIMOC) commenced during late 2017, implementation did not begin until January 2018. Therefore, no admissions during 2017 were managed within the BIMOC service framework. Admissions from January 2018 onwards were managed within the BIMOC service framework and were therefore classified as belonging to the BIMOC service-framework cohort. Data from 2019–2021 were unavailable due to a transition in EMR and reporting systems, including changes to the naming and reporting configuration of the inpatient unit. Despite efforts to retrieve these historical records, complete datasets for this period could not be recovered. No sampling or recruitment procedures were undertaken; all eligible admissions with available EMR and outcome data were included in the analysis.

Because this was a retrospective audit of routinely collected EMR data and service-level KPIs, no a priori sample size calculation was performed. In line with common practice in EMR-based observational studies of child and adolescent mental health services, the focus was on including the full available cohort rather than prospectively determining a required sample [25,26].

### 2.5. Procesure and Data Collection

Data for this study were collected retrospectively across the study period January 2016 to December 2018 and January 2022 to December 2025. Clinical information was originally recorded by treating clinicians as part of usual routine care and maintained within the NSW Health EMR systems, primarily the EMR Discern Analytics reporting tool and the NSW Health reporting platform, Power Business Intelligence (BI) (Mental Health Inform). For the purposes of this study, two clinical researchers (AT and IB) extracted data from EMR Discern Analytics and the NSW Health Power BI reporting platform by authorized personnel with approved access to the relevant systems and verified the data. Then, data were de-identified before analysis and stored on secure password-protected NSW Health systems in accordance with the ethical and data governance requirements. The study collected demographic information, routinely recorded clinical outcome measures, and service-level KPIs. Detailed descriptions of the variables and instruments are provided in Section 2.6.1 and Section 2.6.2.

### 2.6. Measures

#### 2.6.1. Socio-Demographic and KPI Measures

Socio-demographic variables included gender (male, female, others), age (10–12 years, 13–18 years), and ethnicity (Aboriginal, Caucasian, and Culturally and Linguistically Diverse [CALD]). KPIs included length of stay, 28-day readmission, 7-day follow-up completed, seclusion, suicide/self-harm incidents and total admissions. The choice of these measures and KPIs was based on the extensive review of existing studies [12,27,28,29] and clinical relevance. Service-level KPIs, as described above, were compared across models.

#### 2.6.2. Outcome Measures

The outcome measures were completed on admission and discharge using the routinely collected measures within CAMHS services and were recorded in EMR systems. Measures were completed by a clinician who had previously received training in administering these instruments. HoNOSCA and CGAS were selected because they assess two critical dimensions of clinical outcomes: clinical severity and functional impairment through clinician ratings.

##### Health of the Nation Outcome Scale for Children and Adolescents (HoNOSCA)

The HoNOSCA is a clinician-rated instrument for people under 18 years of age, comprising of 15 items covering clinical and psychosocial functioning, including behavior, clinical symptoms, social problems, information problems, and impairment [16]. The total HoNOSCA score ranges from 0 to 52, with higher scores indicating greater symptom severity and psychosocial impairment and lower scores indicating fewer difficulties and improved clinical functioning. Consequently, reductions in HoNOSCA scores over time reflect clinical improvement. This was used in this study as the primary measure of psychopathology.

##### Clinical Global Assessment Scale (CGAS)

The CGAS is a rating of functioning for children and young people aged 6–17 years [17]. The child is given a single score between 1 and 100, based on a clinician’s assessment of various aspects of the child’s psychological and social functioning. Scores fall into 10 categories, ranging from’ extremely impaired’ (1–10) to ‘doing very well’ (91–100). This measure was used as the primary measure of functioning.

### 2.7. Data Analysis

The data were analysed in three stages using the Statistical Package for Social Sciences (SPSS, version 25). Continuous variables (HoNOSCA, CGAS, length of stay, etc.) were summarised using means and standard deviations (SDs), while categorical variables (gender, age groups, ethnicity, etc.) were summarised using frequencies and percentages. First, descriptive statistics were used to summarise sample demographics and assess comparability between the TMOC and BIMOC. Second, the KPIs were examined across these MOCs using descriptive statistics. Finally, clinical outcome measures (HoNOSCA and CGAS) were analysed using paired-samples *t*-tests to evaluate pre–post changes within each model. Effect sizes (Cohen’s d) were calculated to assess the magnitude of change from admission to discharge. Between-model comparisons of effect sizes were conducted using z-tests to examine differences in the magnitude of improvements between the TMOC and BIMOC. Normality assumptions for continuous variables were assessed using a visual inspection of histograms and Q–Q plots for admission and discharge scores, supplemented by Shapiro–Wilk tests where appropriate. These checks indicated approximate normality and supported the use of parametric tests. Comparability between cohorts at baseline was evaluated descriptively by comparing distributions of demographic characteristics (gender, age group, ethnicity) and key clinical variables across the TMOC and BIMOC.

### 2.8. Ethics

This project received ethical approval from the Human Research Ethics Committee (HREC) of the South Western Sydney Local Health District (reference number: 2018/ETH00719). The HREC granted a waiver of informed consent from parents/carers and/or assent from young people since this project involved no more than low risk and analyzed de-identified routinely collected EMR data obtained during standard clinical care. As the BIMOC formed part of routine service delivery rather than a research intervention, no additional treatment procedures were introduced for research purposes. The current study was conducted in accordance with the Declaration of Helsinki [30].

## 3. Results

### 3.1. Demographic Characteristics of Participants

Table 1 presents the demographic profile of admitted young people in the TMOC cohort and the BIMOC service-framework cohort over 2016–2018. Females consistently accounted for the majority of admissions (approximately 67–73%), with a smaller proportion of males (approximately 27–33%) and a minimal representation from other gender categories. Most adolescents were aged 13–18 years (over 92% in all years), indicating that inpatient admissions predominantly comprised adolescents. In terms of ethnicity, Caucasian adolescents constituted the largest group across all years, although there was some variation, with a lower proportion observed in 2017 (68.8%) compared to 2016 and 2018 (~80%). The proportion of Aboriginal adolescents remained relatively stable (approximately 7–8%).

In terms of ethnicity, Caucasian adolescents constituted the largest group across all years, although there was some variation, with a lower proportion observed in 2017 (68.8%) compared with 2016 and 2018 (81.6%). The proportion of Aboriginal adolescents remained relatively stable (approximately 7–8%). Overall, the demographic profile of admitted adolescents was broadly similar across the study years.

The total number of admissions increased from 114 in 2016 and 154 in 2017 under TMOC to 184 in 2018 under the BIMOC. As admission counts reflect service activity rather than demographic characteristics, these figures are presented separately.

### 3.2. Sustained Improvements in Selected KPIs Following BIMOC Implementation

Table 2 shows the comparison of KPIs across the TMOC (2016–2017) and the BIMOC service-framework cohort (2018 onwards), demonstrating differences in KPI performance observed during the TOMOC and BIMOC periods. In 2018, the initial year of the BIMOC being used, there were marked reductions in the average length of stay (13.6 days vs. 21.0 days in 2016), 28-day readmission rates (8.1% vs. 11.9–17.0%), and seclusion events (4.6 vs. 13.8–20.1 per 1000 occupied bed days), alongside a substantial increase in 7-day follow-up completion (76.8% vs. ~60%). These differences were observed alongside an increase in service capacity (admissions), with total admissions rising from 114–154 under the TMOC to 184 in 2018. Suicide and self-harm incidents also decreased initially (101 in 2018 compared to 141 in 2017) and continued to decline over time, reaching 12 incidents by 2025, although these figures represent combined inpatient and community events. In subsequent years (2022–2025), improvements were largely sustained, particularly in follow-up rates (68.9–90.0%) and seclusion events (2–7 per 1000 bed-days), while total admissions remained consistently higher than TMOC levels despite some fluctuations (123–138 admissions per year). Overall, higher service throughput, lower seclusion rates, lower suicide/self-harm incidents, and improved follow-up rates were observed during the BIMOC period, with several of these differences remaining evident over time.

### 3.3. Changes in Clinical Outcomes and Comparative Effectiveness of Models of Care

Across both the TMOC cohort and BIMOC service-framework cohort, statistically significant improvements were observed in clinical outcomes from admission to discharge. Under the TMOC, CGAS scores increased and HoNOSCA scores decreased significantly, indicating improvements in functioning and symptom severity. Similarly, under the BIMOC, substantial improvements were observed across both acute and crisis subgroups, with significant increases in CGAS scores and reductions in HoNOSCA scores. Effect size analysis demonstrated a greater magnitude of improvement in functioning under the BIMOC service-framework cohort (d = 1.89) compared with the TMOC cohort (d = 1.22). This difference was statistically significant (z = −3.694, *p* = 0.0002). However, baseline CGAS scores were lower in the BIMOC service-framework cohort (27.67) than in the TMOC cohort (34.16), indicating poorer functional status at admission and potentially greater scope for improvement. In contrast, improvements in HoNOSCA scores were observed under both models, and between-model comparisons did not identify a statistically significant difference in symptom improvement (z = −0.769, *p* = 0.4418) (Table 3).

## 4. Discussion

This study addresses a key gap in the peer-reviewed literature by comparing different inpatient MOCs using both clinical outcome measures and service-level KPIs within a single Australian service setting. Overall, the findings suggest that the BIMOC was associated with maintained clinical outcomes alongside differences in several service-level indicators, including follow-up rates, seclusion events, and service throughput. Our findings contribute to the emerging evidence base on the effectiveness and sustainability of inpatient MOCs for adolescents globally and within the Australian context.

The current study indicated that the implementation of the BIMOC service-framework maintained clinical effectiveness and contributed to larger observed improvements in functional outcomes compared with the TMOC. However, the BIMOC service-framework cohort demonstrated poorer baseline functional status at admission than the TMOC cohort, which may have provided a greater opportunity for improvement. Accordingly, although larger improvements in functioning were observed during the BIMOC service-framework period, the contribution of baseline differences cannot be excluded, and these findings should be interpreted cautiously. Improvements in symptom severity were observed under both models; however, between-model comparisons did not identify a statistically significant difference in HoNOSCA outcomes. In addition, favorable patterns were observed across several service-level indicators during the BIMOC period, including initial reductions in the length of stay and readmission rates, together with increased service throughput, without evidence of poorer clinical outcomes. These findings are particularly important within acute adolescent mental health inpatient units in Australia, which play a critical role in managing high-risk presentations [31] and face ongoing pressures related to limited bed capacity and increasing demand [32]. The observed efficiency gains suggest that structured, time-limited MOCs may facilitate timely access to inpatient services while maintaining clinical safety and effectiveness in crisis settings [33,34]. Our findings are consistent with the previous literature, indicating that similar models, such as the Rapid Stabilization Pathway [35], have reported shorter lengths of stay without increases in readmission rates, enabling improved access to limited inpatient resources [36]. However, the broader literature also highlights variability in outcomes, with some studies reporting persistent challenges in reducing readmission rates due to clinical complexity and post-discharge factors [37,38]. The comparatively favorable outcomes observed in the current study may reflect the integrated and responsive design of the BIMOC, including enhanced follow-up and continuity of care, which are known to influence service utilization and relapse risk [38]. Importantly, no major changes in ward infrastructure, staffing levels, admission criteria, or service configuration occurred during the study period, apart from the introduction and ongoing implementation of the BIMOC. This supports the likelihood that the observed improvements were associated with the BIMOC rather than major organizational changes within the inpatient service. Overall, these findings contribute to the growing literature supporting innovative MOCs that can address both clinical and operational demands in acute adolescent mental health services [39,40,41].

Our findings also indicated that several of these improvements were sustained over time, particularly higher follow-up rates, reduced seclusion and self-harm incidents, and consistently increased admission capacity, supporting the model’s long-term viability. However, the long-term sustainability of clinical outcomes could not be evaluated because CGAS and HoNOSCA data were only available for the initial implementation period (2016–2018). These findings are broadly consistent with existing literature, which demonstrates that system-level and organizational interventions can produce sustained reductions in restrictive practices, particularly when models prioritize relational, patient-centered care [42]. Moreover, the sustained increase in admission capacity observed in our study aligns with evidence from crisis stabilization and brief inpatient models, which have shown that efficient, structured care pathways can improve patient flow and optimize the use of limited inpatient beds without compromising outcomes [35,42]. However, the existing literature also demonstrates variability in the sustainability of such improvements, with literature reporting challenges in maintaining gains over time due to workforce constraints, service fragmentation, and increasing clinical complexity among admitted adolescents [13]. The sustained benefits observed in the current study may, therefore, reflect the structured design and embedded continuity mechanisms within the BIMOC, including enhanced follow-up processes and consistent service integration, which may mitigate some of these challenges.

The current study’s findings suggest that the BIMOC may have supported a shift toward less coercive practices (e.g., seclusion events) and more patient-centered care (less self-harm incidents), consistent with the contemporary trauma-informed care principles in adolescent mental health services [42,43]. These findings align with the existing literature, demonstrating that system-level and organizational interventions can significantly reduce the use of restrictive practices such as physical restraints and seclusions [44]. Similarly, a systematic review by Kelly et al. [42] found that trauma-informed interventions and service redesign strategies were consistently associated with reductions in seclusion and restraint in youth mental health settings. These studies support the view that changes in care models, particularly those focusing on relational care, staff training, and organizational culture, can effectively reduce reliance on coercive interventions. Overall, the present findings contribute to the growing evidence that innovative models of care can reduce restrictive practices while maintaining patient safety in high-risk adolescent populations [26,45].

Additionally, the present study identified the observed downward trend in suicide and self-harm incidents over time, suggesting potential improvements in risk management and continuity of care. This is clinically and operationally significant in acute child and adolescent mental health inpatient settings. Clinically, this may reflect enhanced engagement, more effective safety planning, and enhanced therapeutic alliance [46]; operationally, it indicates a more stable ward environment with fewer behavioral escalations requiring intensive intervention [47]. These findings are consistent with emerging evidence, demonstrating that structured, brief, and continuity-focused interventions can reduce suicidal behaviors and rehospitalization risk. For example, Goldstein and colleagues [48] reported that a brief post-discharge safety intervention was associated with lower rates of rehospitalization and suicidal behavior among adolescents, highlighting the importance of continuity of care [49]. In contrast, other studies have suggested that self-harm and suicide-related behaviors remain persistent challenges in inpatient settings, particularly in populations with complex comorbidities and limited access to follow-up care [13]. The divergence between these findings and the current study may be explained by differences in MOC structure. Overall, the sustained reduction in suicide and self-harm incidents observed in this study provides important evidence that structured, coordinated models of care can improve both immediate safety outcomes and longer-term clinical stability in high-risk adolescent populations.

### 4.1. Implications for Policy and Clinical Practice

The current study’s findings highlight the importance of developing effective and sustainable inpatient MOCs for young people. The BIMOC service-framework period was associated with maintained clinical effectiveness alongside greater improvements in functional outcomes, while achieving sustained improvements in key service-level indicators, including reduced critical incidents, optimized follow-up, and increased service throughput over time. These results highlight the value of models that integrate clinical effectiveness with operational efficiency and sustainability [40]. From a policy perspective, the findings support the need for adolescent-focused, trauma-informed, and developmentally appropriate care frameworks, as well as stronger integration among inpatient, community, child protection, and educational services to ensure continuity of care. This recommendation is consistent with international mental health policy frameworks, including the World Health Organization’s Comprehensive Mental Health Action Plan 2013–2030 and guidance on adolescent mental health, which emphasize integrated, person-centered, community-based services, continuity of care, early intervention, and developmentally appropriate mental health supports for children and adolescents [2,50]. Improved follow-up rates and reduced restrictive practices reinforce the importance of coordinated care pathways and transition planning.

At a systems level, increased (safe) service capacity without additional financial investment in a setting under constant pressure suggests that service redesign can improve efficiency while maintaining equity and access, which is critical in high-demand settings. Clinically, structured, goal-directed, and family-inclusive approaches may enhance engagement and safety, with potential to reduce self-harm risk. Future policy should prioritize workforce development, multidisciplinary collaboration, and routine outcome monitoring to sustain improvements and support the delivery of high-quality care for young people requiring inpatient mental health services.

### 4.2. Strengths, Limitations and Directions for Future Research

A key strength of this study is the use of clinician-rated outcome measures (HoNOSCA and CGAS) alongside service-level KPIs over multiple years, which allowed for the evaluation of clinical and operational outcomes. To our knowledge, no study has been conducted within the adolescent inpatient setting in Australia, comparing the effectiveness of MOCs using KPIs and psychometric measures. However, the findings should be interpreted in light of several limitations. First, demographic data for the period 2022–2025 and KPI data for 28-day readmissions were incomplete. In addition, the study only used clinical outcome data (HoNOSCA and CGAS), and these were available only for 2016–2018 due to resource constraints and limitations in data access. The incomplete availability of some long-term data reduces the ability to assess all outcomes consistently across the full study period. An additional limitation is the absence of data from 2019 to 2021. This gap resulted from a statewide transition in electronic reporting systems and associated changes in the naming and configuration of the inpatient unit, which prevented the retrieval of complete historical records for those years. Although the BIMOC remained unchanged during this period and no major changes occurred in infrastructure, staffing levels, admission criteria, or service delivery, the absence of these data limits the ability to examine service performance continuously across the entire implementation period. Furthermore, the influence of factors occurring during this period, including the COVID-19 pandemic, could not be directly evaluated. Consequently, while the sustainability of several service-level KPIs could be evaluated through 2025, the long-term sustainability of clinical outcomes (CGAS and HoNOSCA) could not be assessed beyond the initial BIMOC implementation period.

These limitations, including the absence of data from 2019 to 2021, were primarily driven by a statewide transition in reporting platforms, including changes associated with relocation and not using the same data system for the inpatient unit in 2022, which restricted the retrieval of historical data. Consequently, complete datasets for the entire study period could not be accessed retrospectively, despite efforts by the Mental Health Information Management team to retrieve them. Additionally, CGAS data were not available through the Discern Analytics system and would have required manual extraction from clinical records, which was not feasible within available resources. Moreover, the quasi-experimental before-and-after design limits causal inference. Accordingly, observed differences between the TMOC and BIMOC periods should be interpreted as associations rather than definitive evidence that BIMOC alone caused the observed changes. Also, the study relied on a historical comparison between different cohorts admitted during different time periods and did not include a concurrent control group. Consequently, although differences were observed between the TMOC and BIMOC periods, these differences cannot be attributed exclusively to the BIMOC, and the potential influence of unmeasured temporal, organizational, or patient-level factors cannot be excluded. Furthermore, the quasi-experimental design remains susceptible to history effects and temporal confounding. Although no major changes in ward infrastructure, staffing levels, admission criteria, or service delivery model occurred during the study period apart from the implementation of the BIMOC, the potential influence of unmeasured external factors, including broader health system changes, community service developments, and societal events, such as the COVID-19 pandemic, cannot be completely excluded. Importantly, the study was not designed as an equivalence or non-inferiority trial; therefore, the absence of statistically significant differences between models should not be interpreted as evidence of clinical equivalence. A further limitation relates to the differences in baseline functional status between cohorts. Adolescents in the BIMOC service-framework cohort had lower CGAS scores at admission than those in the TMOC cohort, indicating poorer functioning at baseline and potentially greater opportunity for improvement. Because baseline-adjusted analyses (e.g., ANCOVA or group × time interaction models) were not feasible using the available retrospective dataset, the possibility that baseline differences influenced the magnitude of observed functional improvement cannot be excluded. Consequently, comparisons of effect sizes between cohorts should be interpreted cautiously. Future research using prospective non-inferiority or equivalence designs may help establish whether the BIMOC achieves clinical outcomes equivalent to those of traditional inpatient models of care. In addition, prospective studies should also aim to incorporate a broader range of psychometric measures and ensure more comprehensive and consistent data capture over extended time periods, particularly by addressing gaps in demographic, clinical, and KPI data. Efforts to improve data accessibility and integration across evolving reporting systems, alongside designs that strengthen causal inference, would enhance the robustness of evaluations of service effectiveness and sustainability.

## 5. Conclusions

This study suggests that a structured inpatient MOC (BIMOC service-framework) incorporating multidisciplinary collaboration, clearly defined clinical pathways, family involvement, and coordinated discharge and follow-up processes may support positive clinical and service-level outcomes for adolescents admitted to acute mental health inpatient services. Compared with the TMOC period, the BIMOC service-framework period was associated with maintained clinical effectiveness and larger observed improvements in functional outcomes. However, baseline differences between cohorts mean that these findings should be interpreted cautiously and do not establish that the BIMOC itself produced superior functional outcomes. Moreover, given the observational before-and-after design, the findings should be interpreted cautiously and do not establish causality. Also, clinical effectiveness was demonstrated during the initial implementation phase of the BIMOC; however, the long-term sustainability of clinical outcomes could not be confirmed because CGAS and HoNOSCA data were unavailable after 2018. Overall, this study contributes to the emerging evidence base on innovative adolescent inpatient models of care and highlights the potential value of structured, coordinated, and family-inclusive approaches for addressing the clinical and operational demands of contemporary mental health services, particularly within the Australian context.

## Figures and Tables

**Table 1:** Demographic characteristics of the participants admitted under the TMOC and BIMOC.

MOC by Year	Total Admissions	Gender n (%)			Age n (%)		Ethnicity n (%)		
		Male	Female	Others	10–12 Years	13–18 Years	Aboriginal	Caucasian	CALD
TMOC									
2016	114	37 (32.5)	77 (67.5)	0 (0.0)	7 (6.1)	107 (93.9)	8 (7.0)	93 (81.6)	13 (11.4)
2017	154	41 (26.6)	112 (72.7)	1 (0.6)	5 (3.2)	149 (96.8)	13 (8.4)	106 (68.8)	35 (22.7)
BIMOC									
2018	184	56 (34.4)	124 (67.4)	4 (2.2)	14 (7.6)	170 (92.4)	14 (7.6)	147 (79.9)	23 (12.5)

Note: Demographic data from 2022–2025 are not available in Power BI.

**Table 2:** KPI comparison between TMOC and BIMOC.

MOC by Year	Average Length of Stay (Days)	28-Day Readmission Rate	7-Day Follow-Ups Completed	Seclusion *	Suicide/Self-Harm Incidents **	Total Number of Admissions
TMOC						
2016	21.0	11.9	61.9	13.8	87	114
2017	13.9	17.0	59.2	20.1	141	154
BIMOC						
2018	13.6	8.1	76.8	4.6	101	184
2022	22.4	-	90.0	7	42	130
2023	16.6	13.3	68.9	2	47	138
2024	17.8	12.3	87.7	7	54	123
2025	16.2	6.7	86.6	4	12	123

* Events per 1000 occupied bed days. ** Combined inpatient and community incidents. - No data available in Power BI.

**Table 3:** Changes in clinical outcomes (CGAS and HoNOSCA) from admission to discharge across TMOC and BIMOC: within- and between-model comparisons.

MOC (Year)	CGAS Admission (SD)	CGAS Discharge (SD)	*t*-Test	Effect Size (d, 95% CI)	HoNOSCA Admission (SD)	HoNOSCA Discharge (SD)	*t*-Test	Effect Size (d, 95% CI)
TMOC 2016	37.14 (10.49)	57.60 (11.73)			19.00 (7.65)	9.24 (5.34)		
TMOC 2017	31.67 (13.15)	52.39 (14.09)			17.99 (5.55)	11.58 (4.04)		
TMOC Total	34.16 (12.29)	54.87 (13.23)	t(133) = −14.18, *p* < 0.001	1.22 [1.00–1.45]	18.45 (6.58)	10.57 (4.78)	t(146) = 15.94, *p* < 0.001	1.31 [1.09–1.54]
BIMOC 2018 (Acute)	23.44 (9.91)	48.67 (9.06)			19.82 (6.66)	10.61 (6.24)		
BIMOC 2018 (Crisis)	30.27 (10.55)	51.54 (8.89)			19.11 (6.72)	9.58 (5.18)		
BIMOC 2018 Total	27.67 (10.79)	49.76 (9.01)	t(144) = −22.74, *p* < 0.001	1.89 [1.62–2.16]	19.30 (6.67)	9.96 (5.62)	t(151) = 17.74, *p* < 0.001	1.44 [1.21–1.67]

Notes: Demographic data from 2022 to 2025 are not available in Power BI. Within-model comparisons (admission vs. discharge) were conducted using paired samples *t*-tests and are presented in the main body of the table. Between-model comparisons were conducted using z-tests by comparing the magnitude of change (Cohen’s d) between the TMOC and BIMOC. For CGAS, the difference in effect sizes was statistically significant (z = −3.694, *p* = 0.0002). For HoNOSCA, the difference in effect sizes was not statistically significant (z = −0.769, *p* = 0.4418). Effect sizes (Cohen’s d) represent magnitude of change from admission to discharge. Between-model comparisons of effect sizes were conducted using z-tests: CGAS (z = −3.694, *p* = 0.0002); HoNOSCA (z = −0.769, *p* = 0.4418). SD: standard deviation.

## Data Availability

Data can be made available from the corresponding author only on request due to ethical and privacy concerns.

## Supplementary Materials

The following supporting information can be downloaded at: https://www.mdpi.com/article/10.3390/healthcare14172837/s1.

## Abbreviations

The following abbreviations are used in this manuscript:

BIMOC	Brief Intervention Model of Care
BI	Business Intelligence
CALD	Culturally and Linguistically Diverse
CAMHS	Child and Adolescent Mental Health Service
CGAS	Children’s Global Assessment Scale
EMR	Electronic Medical Records
FIHS	Factors Influencing Health Status
GKL	Gna Ka Lun
HoNOSCA	Health of the Nation Outcome Scales for Children and Adolescents
HREC	Human Research Ethics Committee
KPIs	Key Performance Indicators
LOS	Length of Stay
MDT	Multidisciplinary Team
MHSO	Mental Health Service Organization
NMHPF	National Mental Health Performance Framework
RANZCP	Royal Australian and New Zealand College of Psychiatry
SD	Standard Deviation
SDQ	Strengths and Difficulties Questionnaire
TMOC	Traditional Model of Care
