# Supplementary material for: Innovation in Acute Adolescent Mental Healthcare: Evaluating a Responsive and Sustainable Mental Healthcare Model
Source: Healthcare (Basel). 2026 Sep 3;14(17):2837. doi: 10.3390/healthcare14172837 (PMC13565297; doi:10.3390/healthcare14172837)
Supplement: Supplementary file 1 [file healthcare-14-02837-s001.zip › healthcare-4407969-supplementary.pdf]

Section S1

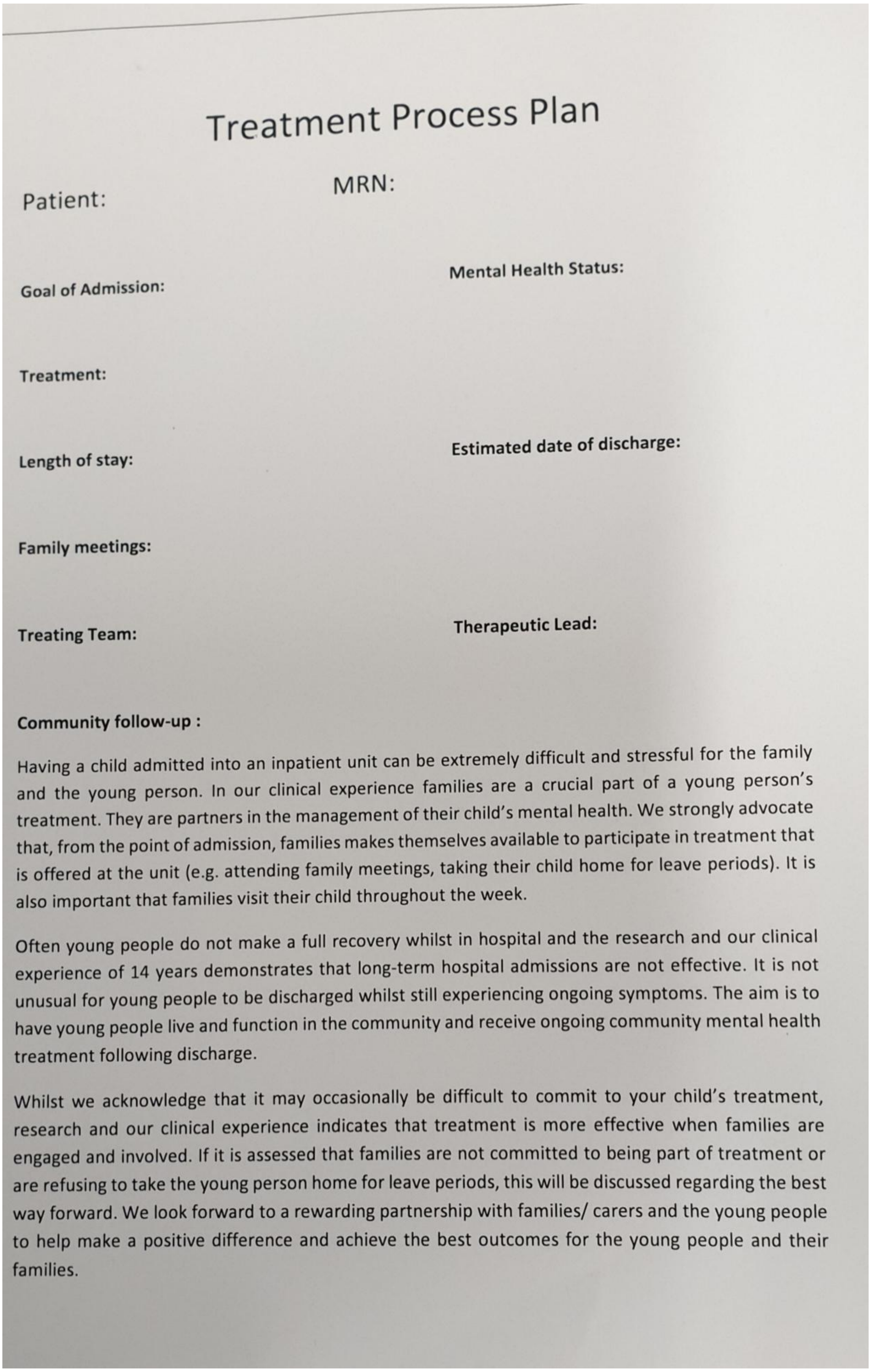

Treatment Process Plan

Patient:

MRN:

Goal of Admission:

Mental Health Status:

Treatment:

Length of stay:

Estimated date of discharge:

Family meetings:

Treating Team:

Therapeutic Lead:

Community follow-up :

Having a child admitted into an inpatient unit can be extremely difficult and stressful for the family and the young person. In our clinical experience families are a crucial part of a young person's treatment. They are partners in the management of their child's mental health. We strongly advocate that, from the point of admission, families makes themselves available to participate in treatment that is offered at the unit (e.g. attending family meetings, taking their child home for leave periods). It is also important that families visit their child throughout the week.

Often young people do not make a full recovery whilst in hospital and the research and our clinical experience of 14 years demonstrates that long-term hospital admissions are not effective. It is not unusual for young people to be discharged whilst still experiencing ongoing symptoms. The aim is to have young people live and function in the community and receive ongoing community mental health treatment following discharge.

Whilst we acknowledge that it may occasionally be difficult to commit to your child's treatment, research and our clinical experience indicates that treatment is more effective when families are engaged and involved. If it is assessed that families are not committed to being part of treatment or are refusing to take the young person home for leave periods, this will be discussed regarding the best way forward. We look forward to a rewarding partnership with families/ carers and the young people to help make a positive difference and achieve the best outcomes for the young people and their families.

Section S2

Brief Intervention Model

Phase 1: With Adolescent

- **Intro - Building Rapport and a positive therapeutic relationship**
- **Set the framework for treatment**

Confidentiality

Feedback of bio-psycho-social formulation in developmentally appropriate terms.

Review of treatment process plan-identify members of the treating team, the therapeutic lead, individual and family sessions, proposed discharge date.

- **Understanding of the Brief Intervention Model**
- **Understanding what led to the crisis and provide a safe space for the admitted adolescent to express themselves.**
- **Begin to develop a Care Plan, focusing on the "My triggers and warning signs", "How can other people help me?", and "How can I feel better?"**
- **Provide the client with psychoeducation**
- Diagnosis and treatment
- The importance of self-care
- Managing Emotions
- Managing Distress

Phase 2: With Adolescent:

Skills training: (over 2-3 sessions)

- Mindfulness
- Distress tolerance
- Emotional regulation

Develop further the Care Plan, focusing on "Strengths" and Supports- family, friends, school

Provide psychoeducation about the development and maintenance of the specific problem

Provide psychoeducation on the benefits of further treatment

Phase 3:

Re-formulation based on improved understanding of the adolescent' history, development and family dynamics.

Phase 4:

- **Reflect**
- **Review**
- **Future goals**
- **Relapse prevention.**

<u>Session with family/carers:</u>

Phase 1:

Build rapport:

Focus on the here-and-now and validate the challenging nature of the carer journey. Acknowledge the carer's efforts to attend the session, and the struggles they experience in their caregiving role.

Collaborate with the family/carer to develop a shared understanding of the adolescents' presenting issues.

Feedback based on biopsychosocial formulation

Psychoeducation:

- Diagnosis
- Treatment
- Introduce the treatment process plan-formalize family meeting sessions.

Phase 2:

Psychoeducation with focus on emotional dysregulation, self-harm and /or high- risk behaviours.

Introduce care-plan developed in collaboration with the admitted adolescent.

Introduce the concept of mindfulness, emotional coaching and distress tolerance skills.

Phase 3:

Explore family dynamics

Parenting style

Communicating patterns.

Phase 4:

Feedback on identified triggers, predisposing and maintaining factors in the adolescents' presentation.

Develop communication strategies so as to empower the family/carers to de-escalate crisis situation and improve inter-personal relationship.

WRAP-UP SESSION:

Joint session involving the treating team, admitted adolescent and family/carers and community team.

Reflect on the admission and treatment process.

Identify the strengths, gains made and barriers to treatment.

Discharge planning- follow-up appointment, setting goals for ongoing treatment in the community, relapse prevention.
